# Action observation treatment-based exoskeleton (AOT-EXO) for upper extremity after stroke: study protocol for a randomized controlled trial

**DOI:** 10.1186/s13063-021-05176-x

**Published:** 2021-03-20

**Authors:** Zejian Chen, Nan Xia, Chang He, Minghui Gu, Jiang Xu, Xiaohua Han, Xiaolin Huang

**Affiliations:** 1 Department of Rehabilitation Medicine, Tongji Hospital, Tongji Medical College, Huazhong University of Science and Technology, Wuhan, China; 2 World Health Organization Cooperative Training and Research Center in Rehabilitation, Wuhan, China; 3 Institute of Rehabilitation and Medical Robotics, State Key Lab of Digital Manufacturing Equipment and Technology, Huazhong University of Science and Technology, Wuhan, China

**Keywords:** Stroke, Action observation treatment, Exoskeleton, Upper limb, Functional magnetic resonance imaging, Randomized controlled trial

## Abstract

**Background:**

Stroke produces multiple symptoms, including sensory, motor, cognitive and psychological dysfunctions, among which motor deficit is the most common and is widely recognized as a major contributor to long-term functional disability. Robot-assisted training is effective in promoting upper extremity muscle strength and motor impairment recovery after stroke. Additionally, action observation treatment can enhance the effects of physical and occupational therapy by increasing neural activation. The AOT-EXO trial aims to investigate whether action observation treatment coupled with robot-assisted training could enhance motor circuit activation and improve upper extremity motor outcomes.

**Methods:**

The AOT-EXO trial is a multicentre, prospective, three-group randomized controlled trial (RCT). We will screen and enrol 132 eligible patients in the trial implemented in the Department of Rehabilitation Medicine of Tongji Hospital, Optical Valley Branch of Tongji Hospital and Hubei Province Hospital of Integrated Chinese & Western Medicine in Wuhan, China. Prior to study participation, written informed consent will be obtained from eligible patients in accordance with the Declaration of Helsinki. The enrolled stroke patients will be randomized to three groups: the CT group (conventional therapy); EXO group (exoskeleton therapy) and AOT-EXO group (action observation treatment-based exoskeleton therapy). The patients will undergo blinded assessments at baseline, post-intervention (after 4 weeks) and follow-up (after 12 weeks). The primary outcome will be the Fugl-Meyer Assessment for Upper Extremity (FMA-UE). Secondary outcomes will include the Action Research Arm Test (ARAT), modified Barthel Index (MBI), kinematic metrics assessed by inertial measurement unit (IMU), resting motor threshold (rMT), motor evoked potentials (MEP), functional magnetic resonance imaging (fMRI) and safety outcomes.

**Discussion:**

This trial will provide evidence regarding the feasibility and efficacy of the action observation treatment-based exoskeleton (AOT-EXO) for post-stroke upper extremity rehabilitation and elucidate the potential underlying kinematic and neurological mechanisms.

**Trial registration:**

Chinese Clinical Trial Registry ChiCTR1900026656. Registered on 17 October 2019.

**Supplementary Information:**

The online version contains supplementary material available at 10.1186/s13063-021-05176-x.

## Background

Stroke is the leading cause of mortality and disability worldwide, placing a significant burden on healthcare facilities and socioeconomic systems [1]. Stroke produces numerous symptoms, including sensory, motor, cognitive and psychological dysfunctions, among which motor deficit is the most common and is widely recognized as a major contributor to long-term functional disability [2, 3]. Upper-extremity motor impairments manifest in loss of fractionated movement, coordination and dexterity, resulting in limitations in activities of daily living (ADL) and social participation [4]. Over the last several decades, various rehabilitation approaches have been developed to promote upper extremity motor recovery, functional performance and quality of life in stroke patients [5, 6]. These novel approaches are commonly predicated on the principles of motor learning and task-specific training to facilitate motor neural network plasticity [7–9].

Robot-assisted training is an innovative exercise-based technique that involves the principles of motor learning, such as highly repetitive, intensive and task-specific training with feedback [10]. Studies have shown that robot-assisted training can improve arm muscle strength and upper extremity motor impairment [11]. However, the results in terms of arm motor function and ADL after stroke remain debatable [12, 13]. To strengthen the effects of robot-assisted training on arm function and to explore the involved neural mechanisms, researchers have integrated numerous different techniques into the robotic training routine (e.g. functional electrical stimulation, transcranial direct current stimulation, virtual reality). As a training platform, robotic devices can be programmed simultaneously with other techniques in the adaptive, motivational and quantifiable training methods for boosting neuroplasticity [14].

Action observation therapy (AOT) has been applied as an alternative or complement to another approach to rebuild motor function for stroke rehabilitation and was developed on the neurophysiological basis of the mirror neuron system (MNS) [15, 16]. Action observation therapy consists of action observation followed by action imitation and execution, considering that both processes share the matching cerebral motor network. During AOT, patients are instructed to carefully observe the video sequences, imitate them and then practice those movements and motor tasks. Studies have indicated that action observation would induce neural plasticity by promoting activation of the damaged motor circuits that enhance training effects involved in motor learning after stroke [17–19].

Although there have been extensive articles investigating the application of robotic devices and action observation therapy in upper limb rehabilitation after stroke, they fail to show a superior effect in arm function and ADL compared with conventional therapy. Moreover, according to our knowledge, few studies have examined the combined effects and underlying mechanisms of these two techniques. We hypothesize that action observation treatment coupled with robot-assisted training may enhance motor circuit activation and improve upper extremity motor outcomes. Therefore, in this study, we aim to evaluate the effects of action observation treatment-based exoskeleton (AOT-EXO) treatment for the upper extremity after stroke and to explore the potential kinematic mechanisms assessed by inertial measurement unit (IMU) and neurological mechanisms using functional magnetic resonance imaging (fMRI) and corticospinal excitability metrics.

## Methods

### Study design

This protocol follows the Consolidated Standards of Reporting Trials (CONSORT) Statement on randomized trials of non-pharmacological treatment. The study will be reported according to the SPIRIT (Standard Protocol Items: Recommendations for Interventional Trials) statement (Additional file 1). The Clinical Trials Ethics Committee of Huazhong University of Science and Technology provided ethical approval on 24 October 2018. The trial was registered in the Chinese Clinical Trial Registry, ChiCTR1900026656, on 17 October 2019.

**Fig. 1:**
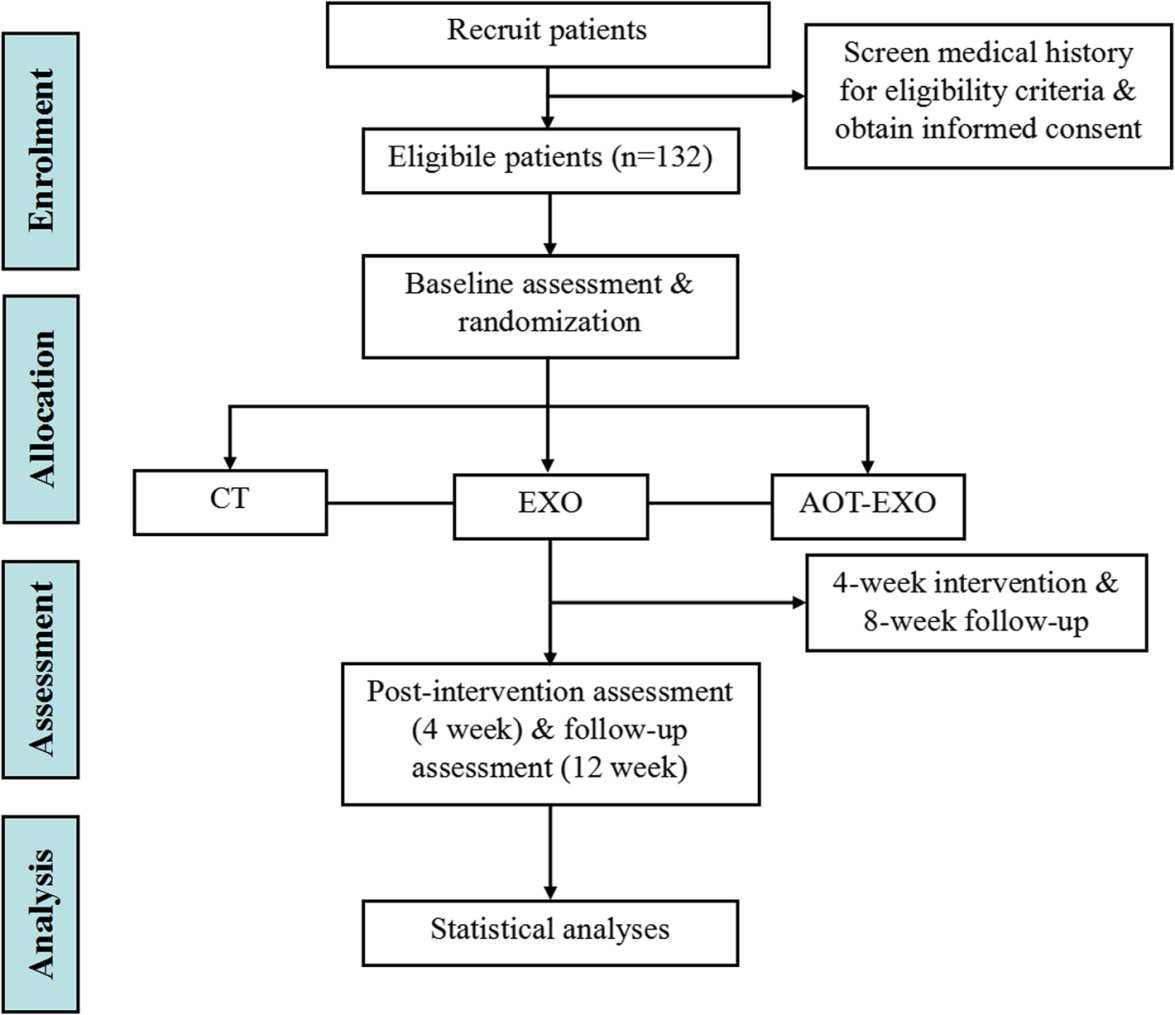
Study flow diagram

**Fig. 2:**
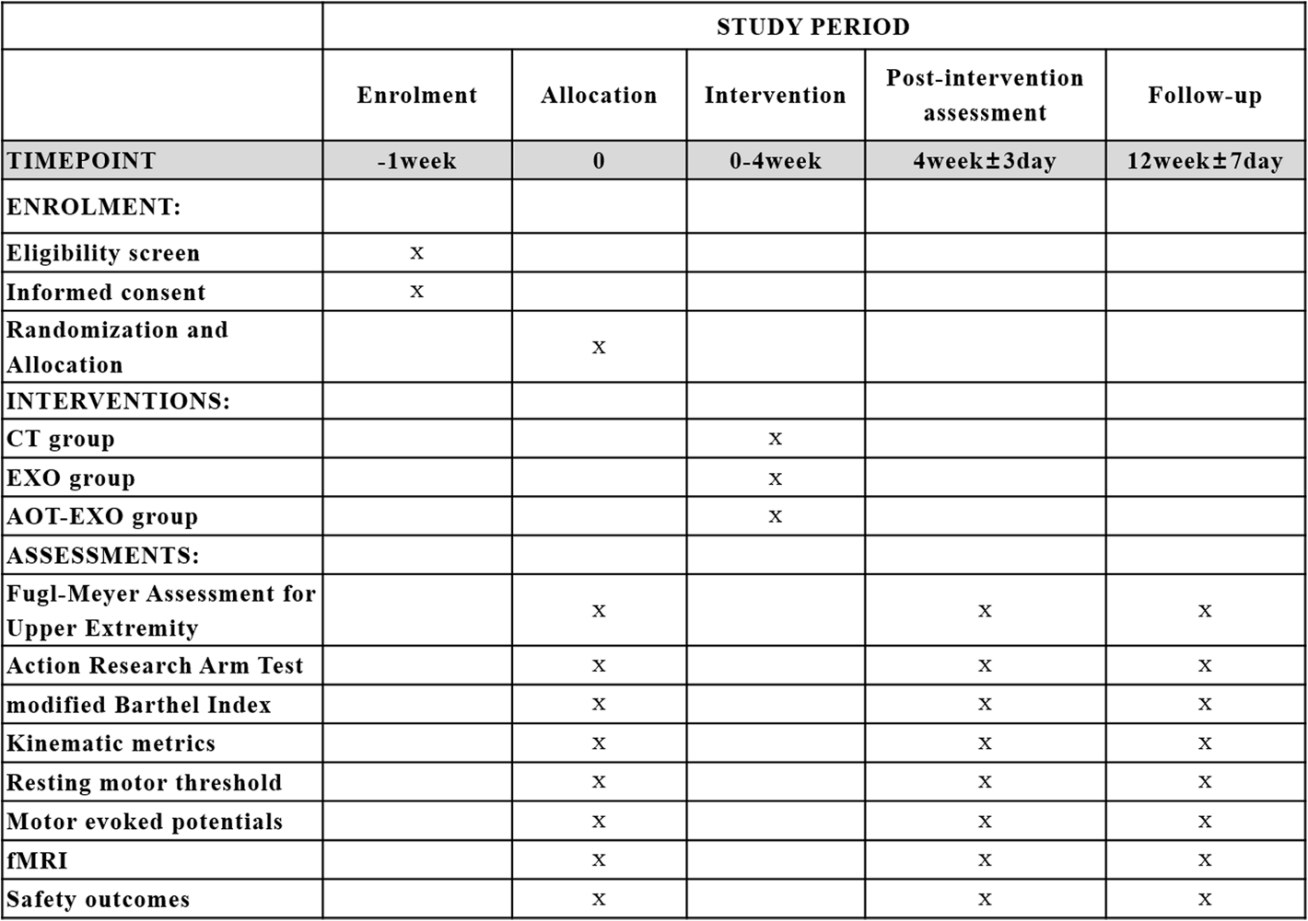
Standard Protocol Items: Recommendations for Interventional Trials (SPIRIT)

The AOT-EXO trial is a multicentre, prospective, three-group randomized controlled trial (RCT). It will be implemented in the Department of Rehabilitation Medicine of Tongji Hospital, Optical Valley Branch of Tongji Hospital and Hubei Province Hospital of Integrated Chinese & Western Medicine in Wuhan, China. A flowchart overview of the study is presented in Fig. 1. The Standard Protocol Items: Recommendations for Interventional Trials (SPIRIT) for enrolment, interventions and assessments is presented in Fig. 2.

Prior to study participation, written informed consent (Additional file 2) will be obtained from eligible patients in accordance with the most recent Declaration of Helsinki. In total, 132 eligible subjects will be randomly allocated into three groups equally: (1) CT group (conventional therapy), (2) EXO group (exoskeleton therapy) and (3) AOT-EXO group (action observation treatment-based exoskeleton therapy). All subjects will receive assessments at baseline, post-intervention (after 4 weeks) and follow-up (after 12 weeks). To avoid assessment bias, an independent evaluator at each centre blinded to the randomization procedure and group allocation will complete all outcome measures.

### Participants

The inclusion criteria are as follows: (1) age 18–80, (2) clinical diagnosis of stroke (cerebral infarction, primary intracerebral haemorrhage, subarachnoid haemorrhage) within 6 months before enrolment, (3) Fugl-Meyer Assessment of the Upper Extremity (FMA-UE) score 8–47, (4) Mini-Mental State Examination (MMSE) score ≥ 22 and compliance with the interventions and (5) provision of written informed consent [20, 21].

The exclusion criteria are as follows: (1) more than one stroke (patients with previous transient ischaemic attack could participate); (2) other current significant impairment of the upper limb, e.g. fixed contracture, shoulder subluxation, or recent fracture; (3) severe visual deficits or unilateral spatial neglect; (4) diagnosis likely to interfere with rehabilitation or outcome assessments, e.g. traumatic brain injury, meningitis and epilepsy; and (5) current participation in another upper limb rehabilitation trial [21, 22]. Of note, patients with contraindications to transcranial magnetic stimulation and functional magnetic resonance imaging (e.g. pacemakers, claustrophobia, implanted metal, implantable electronic devices) will NOT be excluded but will NOT receive corticospinal excitability or fMRI examination.

### Randomization and blinding

The following information will be collected: demographic information, medical history, medication and rehabilitation details, stroke type, time post stroke onset, complications, comorbidity and baseline outcome measures.

We will randomize all the included patients into the three groups at a ratio of 1:1:1 through a computer-generated random number table centrally generated in Tongji Hospital. An independent statistician will prepare the sequentially numbered, opaque, sealed envelopes before the start of enrolment. No randomization number will be reutilized in any case. Because the training methods differ between groups and according to the features of the robot, it is not feasible to blind the subjects, therapists or physicians participating in the trial. Therefore, an independent evaluator at each centre blinded to the randomization procedure and group allocation will assess all outcome measures (baseline, post-intervention and follow-up measures).

### Intervention

Stroke patients in all groups will require multidisciplinary medication and rehabilitation; hence, their routine treatment programmes will continue as usual with the additional 4-week intervention. The intervention will be delivered at the same frequency and duration in the three groups: 45 min daily, 5 days/week for 4 weeks. The same therapists and therapy assistants will deliver all the intervention sessions at each centre [12].

Participants in the CT group (conventional therapy) will receive the therapist-mediated arm intervention using conventional therapy techniques, such as muscle strengthening, stretching, arm exercises or gross-motor training, fine-motor training and functional task practice. This therapy matches that in the other groups with respect to schedule and the form and intensity of movements. The programmes in the conventional group will progress to meet the patient’s training goals.

The EXO group (exoskeleton therapy) will receive intensive task-specific training in the three-dimensional workspace of the human arm involving shoulder (flexion/extension, horizontal abduction/adduction, external/internal rotation) and elbow (flexion/extension, pronation/supination) movements (Armule®, Intelbot Intelligent Machine Co., Ltd., Wuhan, China). Each training session consists of exercises in the following order: 5-min passive mode plus 15-min assist-as-needed mode for twice with a 5-min break between intervals. In the passive mode, the exoskeleton manipulates the upper limb in a three-dimensional trajectory predetermined by the therapists according to the patient’s goals. In the assist-as-needed mode, the patients practice games and ADL training programmes dedicated to shoulder and elbow movements. When the patient is unable to complete the tasks actively, assistive force will supply the upper extremity with passive movement to the target positions.

The AOT-EXO group (action observation treatment-based exoskeleton therapy) will share a training routine with the EXO group. The difference is as follows: during the assist-as-needed session in the AOT-EXO group, patients will be instructed to carefully watch video clips showing upper limb movements and related daily routine tasks before training. Each session will last approximately 15 min (2-min action observation and 3-min action execution for three movement sequences) twice per day (upper limb movements followed by related daily routine tasks in the second session). The actors in the videos will be healthy volunteers. The video clips will be obtained with a Canon camera in the first-person perspective [23]. Patients will be presented with three movement sequences per week for 4 weeks with increasing difficulty (12 sequences in total).

### Outcomes

All outcomes will be measured at baseline, post-intervention (at 4 weeks) and follow-up (at 12 weeks) with no charge. The primary outcome will be mean change in the Fugl-Meyer Assessment for Upper Extremity (FMA-UE) score. This assessment examines arm movement, coordination and reflexes, and the results are represented as a 3-point ordinal scale score with a maximum of 66 points. The FMA-UE is a reliable and valid scale with which to measure upper limb motor impairment after stroke, with higher scores indicating better motor function [24].

Secondary outcomes will be the mean change in Action Research Arm Test (ARAT) score, modified Barthel Index (MBI), kinematic metrics, resting motor threshold (rMT) and motor evoked potentials (MEP), functional magnetic resonance imaging (fMRI) and safety outcomes.

The Action Research Arm Test (ARAT) evaluates 19 tests of arm motor function, including four subscales: grasp, grip, pinch and gross movement. Each test is given an ordinal score of 0, 1, 2 or 3, with the total score ranging from 0 to 57, with higher values indicating better arm motor status and dexterity [25].

The modified Barthel Index (MBI) is an assessment tool formulated to examine the level of independence in the basic ADL and comprises 10 categories: feeding, personal hygiene, toilet use, bladder control, bowel control, bathing, dressing, chair/bed transfer, ambulation, and stair climbing [26].

We will use kinematic metrics (Inertial Measurement Unit, IMU, Noraxon USA Inc.) to evaluate the movement smoothness (peak speed, time to peak speed, number of movement units and path ratio) and performance (pointing time and pointing accuracy) during the finger-to-nose test. The instrumented kinematic assessment can provide good sensitivity for detecting motor performance that is not captured by the clinical scales [27, 28].

Corticospinal excitability will be measured using resting motor threshold (rMT) and motor evoked potentials (MEP) from the abductor pollicis brevis (APB) muscles [29]. Patients will be instructed to sit in a comfortable chair with their hands resting on their lap during the evaluations. Transcranial magnetic stimulation (TMS MagVenture® MagPro R30 Denmark) will be performed using a figure-eight coil placed tangentially in a posterior–anterior plane at a 45° angle from the midline over the hotspot. The rMT is defined for each hemisphere as the minimal stimulator output intensity that elicits MEPs with peak-to-peak amplitudes of at least 50 μV from the contralateral APB muscle in at least five of ten trials. MEPs will be sampled during TMS at 110% rMT and will be kept constant during the following evaluations. The MEP will be recorded as zero if not evocable [30, 31].

Functional magnetic resonance imaging (fMRI) acquisition and pre-processing will be conducted as follows. Scanning will be on the same 3.0 Tesla MR scanner (Discovery LS MR 750; GE Healthcare, Chicago, IL, USA), including acquisition of high-resolution anatomical images, followed by two fMRI series of two hemiplegic-sided motor tasks: finger-to-nose motion (task 1) and finger-to-nose motion sequence observation and execution (task 2). The range of motion for each task is not controlled but rather self-determined. Each series contains 30-s epochs that alternate rest with 0.03-Hz movement. We will acquire functional images using the T2*-weighted gradient echo planer imaging sequence, which measures the blood oxygen level dependence (BOLD) signal. Scanning parameters will include 43 axial 3-mm-thick slices of the whole brain per repetition, 204 volumes/series, TR = 3000 ms and TE = 30 ms, matrix size = 72 × 72 and FOV = 216 × 216 mm^2^. During the fMRI scan, subjects will view a guidance video displayed on the screen. The video will run continuously with a 0.03-Hz movement cycle that is green during rest epochs, yellow during task 1 epochs and red during task 2 epochs. For all conditions, each series will consist of 5 cycles alternating between a 30-s baseline block (fixation cross) followed by a 30-s task block, yielding a total duration of each fMRI run of 10 min. Before entering the scanner, subjects will practice the paradigm and an investigator will observe subject movements during scanning to verify task compliance [32].

Pre-processing of fMRI data will be performed with SPM8 (The Wellcome Department of Imaging Neuroscience, University College, London, UK) running in MATLAB (version 7.9.0.529, R2016b) (The MathWorks, Natick, Massachusetts, USA). For each subject, after motion correction, the anatomical MRI will be co-registered to the mean functional image. The fMRI data pre-processing steps include (1) inter-slice timing correction to the middle slice of a volume for signal coherence, (2) realignment of the motion artefact to the first volume in the entire scanning period, (3) co-registration normalization of spatial transformation to the standardized head domain merged from all subjects’ imaging results (the Montreal Neurological Institute template, called the MNI template) and (4) spatial smoothing with a Gaussian kernel of 4 mm full to half-maximum to elevate the signal-to-noise ratio of functional responses.

Safety outcomes will include the occurrence of all adverse events and serious adverse events in accordance with the guidance of the Clinical Trials Ethics Committee of Huazhong University of Science and Technology. Possible adverse events attributable to the intervention may include shoulder pain, skin abrasion and delayed muscle soreness, which will be observed in detail and documented. Establishing causality between the protocol and adverse events will require judgment. We will rate the relevance degree of all adverse events and the intervention in the AOT-EXO trial with an ordinal score from one to five, with higher values indicating a greater degree of relevance. Although robotic training has been proven safe in the previous studies, serious adverse events may occur related or unrelated to our protocol. We have rehearsed an in-hospital first-aid plan that can be carried out expertly under emergency circumstances and will immediately report to the project investigator together with Ethics Committee, who will determine whether the participant needs to be removed from the study and whether the trial should be adjusted or terminated.

### Sample size

The sample size was calculated by considering the primary outcome measure as the reference: a mean difference of 5 points in the FMA-UE between the AOT-EXO group and CT group and 3 points between the AOT-EXO group and EXO group with a standard deviation of 5.0 [33]. Based on multiple comparisons significance level of 1.67% and a power of 0.8, a sample size of 40 per group will be needed. Assuming that 10% patients loss to follow-up, we will choose a sample size of 44 participants for each group and 132 participants in total. The sample size calculation was performed with PASS 2008 software.

### Data monitoring and management

The data monitoring committee (DMC), independent from the sponsor and competing interests, will be responsible for data monitoring procedure to ensure the accuracy and completeness of the data reported by researchers. The DMC will conduct the procedure at each centre in cooperation with all investigations from institutional review board or regulatory authority. Each centre will be responsible for quality control of the informed consent, participant recruitment, intervention programme implementation and data management.

The directors of each centre will be responsible for retaining all records with complete confidentiality for 10 years after the completion or termination of the study. Each centre will apply data cleansing, identification and coding and will create an electronic data set for statistical analysis and transfer of quality control records. In cases where inconsistencies, missing values or other problems are detected in the data set, the directors at each centre will be queried and will recheck the electronic case report form (CRF) if necessary. The principal investigator and all directors of each centre will receive full anonymous copies of the electronic data set to promote dissemination of the study results.

The sponsor will appoint the Trial Steering Committee (TSC) comprising investigators and clinical experts independent of the clinical trial. TSC will be responsible for the scientific integrity of the clinical trial, including the scientific validity of the study protocol, assessment of study quality and conduct and scientific quality of the final study report.

### Statistical analyses

All statistical analyses will be performed using SPSS software (version 21.0, IBM Corporation, Chicago, IL). To assess the normal distribution of quantitative data, the Shapiro-Wilk test will be employed (*P* > 0.05). Continuous variables will be presented as the mean (SD) for normal distributions and median for non-normal distributions and categorical variables will be presented as frequencies or percentages. Baseline data between groups will be compared using Student’s t-test for continuous variables and the chi-square or Fisher’s exact tests for categorical variables. A *P* value < 0.05 will be considered indicative of statistical significance. All analyses will be carried out based on the intention-to-treat principle, with missing data calculated using multiple imputation. Within-group differences will be assessed using paired t tests for the normally distributed data and Wilcoxon-signed rank test for the non-parametric equivalent test. When comparing the two patient subgroups at each time point, Student’s *t* test (for continuous data) and Fisher’s exact test (for categorical data) will be implemented. When examining the effect of the randomization procedure over time, two-way repeated measures analysis of variance (ANOVA) will be conducted to examine the time-treatment group interaction, with time as the within-group variable and treatment group as the between-group variable.

## Discussion

Previous studies have demonstrated that robot-assisted training is effective in promoting upper extremity muscle strength and motor function after stroke [11]. Additionally, action observation treatment can enhance the effects of physical and occupational therapy by increasing neural activation [16]. However, compared with conventional therapy, the results fail to show a superior effect in arm function and ADL. We hypothesize that action observation treatment coupled with robot-assisted training may enhance motor circuit activation and facilitate upper extremity recovery. Therefore, in this study, we aim to evaluate the effects of AOT-EXO for the upper extremity after stroke and to explore the potential mechanisms underlying its effects.

Several features distinguish this study from previous reports. First, to our knowledge, the AOT-EXO trial is the first clinical trial to integrate robot-assisted training with action observation treatment. As mentioned before, robot-assisted training provides highly intensive and task-specific training based on motor learning theory to boost neural plasticity, while AOT specially primes the mirror neural system to activate motor circuits during training. The combination protocol may exert fortifying effects on post-stroke upper extremity rehabilitation. Second, few previous studies have focused on fMRI and corticospinal excitability changes in stroke patients receiving robot-assisted training. The present study is important for further understanding the neural plasticity mechanisms of motor function recovery to enhance the effectiveness of the present therapies. In addition, the AOT-EXO study will provide extensive kinematic assessments evaluating end-point motor performance and movement smoothness reflecting intersegment coordination.

The present study has some limitations. The exoskeleton in this protocol is very large and not MRI-compatible; thus, it is not possible to evaluate the activation of brain regions using fMRI during therapy. The development of MRI-compatible robots or neurofeedback-based training (e.g. functional near-infrared spectroscopy, electroencephalography, and magnetoencephalography) may allow real-time acquisition of the structural, electrophysiological and metabolic changes in the brain to achieve more satisfactory results during robot-assisted training in the future [34]. Moreover, the ADL-related exoskeleton training is still unable to simulate real ADL. Virtual reality may engage patients, increase their attention and improve motivation during robotic training [35]; hence, we plan to incorporate virtual reality technique in the action observation process to match the ADL contents in the next generation of the present exoskeleton.

In conclusion, this study aims to evaluate the effects of AOT-EXO for the upper extremity after stroke and to explore the potential underlying kinematic and neurological mechanisms. The results of the trial will provide evidence regarding the feasibility and effectiveness of AOT-EXO for post- stroke upper extremity rehabilitation.

### Trial status

Current recruitment. At the time of submission, 12 stroke patients have been enrolled.

Protocol version number V1.0, December 2018. Recruitment began on 18 October 2019, and it is anticipated that this trial will be completed by December 2021.

## Supplementary Information

**Additional file 1.**

**Additional file 2.**

## Data Availability

We aim to publish results derived from this project in international peer-reviewed scientific journals. Data file are available from the corresponding author upon reasonable request.

## Abbreviations

ADL: Activities of daily living
AOT: Action observation therapy
AOT-EXO: Action observation treatment-based exoskeleton
APB: Abductor pollicis brevis
ARAT: Action Research Arm Test
BOLD: Blood oxygen level dependence
CRF: Case report form
DMC: Data monitoring committee
FMA-UE: Fugl-Meyer Assessment of the Upper Extremity
fMRI: Functional magnetic resonance imaging
MBI: Modified Barthel Index
MEP: Motor evoked potentials
MMSE: Mini-Mental State Examination
MNS: Mirror neuron system
RCT: Randomized controlled trial
rMT: Resting motor threshold
TSC: Trial Steering Committee
