# Supplementary material for: Action observation treatment-based exoskeleton (AOT-EXO) for upper extremity after stroke: study protocol for a randomized controlled trial
Source: Trials. 2021 Mar 20;22:222. doi: 10.1186/s13063-021-05176-x (PMC7981809; doi:10.1186/s13063-021-05176-x)
Supplement: Supplementary file 2 — Additional file 2. [file 13063_2021_5176_MOESM2_ESM.doc]

**知情同意书**

**项目名称：上肢机器人联合动作观察治疗对脑卒中上肢运动功能的影响研究**

您将被邀请参加上述康复治疗研究。本知情同意书提供给您一些信息以帮助您决定是否参加此项临床研究。请您仔细阅读，如有任何疑问请向负责该项研究的研究者提出。您参加本项研究是自愿的。

**研究目的：**

上肢机器人辅助康复治疗是新颖、安全、为患者提供足量、以活动为导向的功能性训练，有助于促进上肢力量、协调性恢复以及提高生活自理能力。动作观察疗法有助于激活受损的运动皮质、与相关皮质进行功能重组，有助于对观察动作记忆的强化和运动技能的学习，促进患者上肢运动功能恢复及日常生活能力的提高。尚未有研究显示两者结合治疗效果，故开展本研究探讨上肢机器人联合动作观察治疗对脑卒中上肢运动功能恢复的影响。

**研究过程：**

在治疗前后，将对您的上肢运动功能进行评估、前往门诊楼11楼评估脑诱发电位测量，前往影像科进行fMRI检查，对脑部损伤情况和未来恢复的情况进行评估；您将前往OT治疗室进行上肢康复训练。训练每天进行一次，每次30分钟,一周5天，进行4周。试验过程中，过程中若有任何不适，您可以休息并请及时告诉随同的治疗师。

**隐私问题：**

如果您决定参加本项研究，您有可能被采用上述治疗或常规康复治疗，并进行相关功能评估，本试验所有评估均为免费提供，**参加试验及在试验中的个人资料均属保密**。可以识别您身份的信息将不会透露给研究小组以外成员，除非获得您的许可为。这项研究结果发表时，将不会披露您个人的任何资料。

您可以选择不参加本项研究，或者在任何时候通知研究者要求退出研究，您的数据将不纳入研究结果，您的任何医疗待遇与权益不会因此而受到影响。

如果您需要其它治疗，或者您没有遵守研究计划，或者发生了与研究相关的损伤或者有任何其它原因，项目负责人可以终止您继续参与本项研究。

您可随时了解与本研究有关的信息资料和研究进展，如果您有与本研究有关的问题，或您在研究过程中发生了任何不适与损伤，或有关于本项研究参加者权益方面的问题您可以与主管医生联系。

**知情同意书·同意签字页**

**受试者声明**

我已经仔细阅读了本知情同意书，我有机会提问而且所有问题均已得到解答。我理解参加本项研究是自愿的，我可以选择不参加本项研究，或者在任何时候通知研究者后退出而不会遭到歧视或报复，我的任何医疗待遇与权益不会因此而受到影响。如果我需要其他诊断/治疗，或者我没有遵守研究计划，或者有其他合理原因，研究者可以终止我继续参与本项临床研究。

我自愿参加本次研究，并同意按照知情同意书的内容配合研究医生进行检测，尽力完成本次研究。我将收到一份签过字的“知情同意书”副本。

受试者（签名）：_____________________

联系电话：_____________________日期：___________ 年_____月_____日

受试者因无行为能力等原因不能签署知情同意的，由其法定代理人或监护人签署。

法定代理人或监护人（签名）： __________________

同受试者关系：__________________

联系电话：_____________________日期：___________ 年_____月_____日

**研究者声明**

我已向该受试者充分解释和说明了本临床试验的目的、操作过程以及受试者参加该试验可能存在的风险和潜在的获益，并满意的回答了受试者的所有相关问题。

研究者（签名）：_____________________

联系电话：_____________________日期：___________ 年_____月_____日
